# CD4 T cell deficiency attenuates ischemic stroke, inhibits oxidative stress, and enhances Akt/mTOR survival signaling pathways in mice

**DOI:** 10.1186/s41016-018-0140-9

**Published:** 2018-11-08

**Authors:** Hongfei Zhang, Xiaoxing Xiong, Lijuan Gu, Weiying Xie, Heng Zhao

**Affiliations:** 1 Department of Neurosurgery, Stanford University School of Medicine, 1201 Welch Rd., MSLS Bldg., Room P306, Stanford, CA 94305 USA; 2 Department of Anesthesiology, Zhujiang Hospital of Southern Medical University, Guangzhou, Guangdong China; 3 Department of Neurosurgery, Renmin Hospital of Wuhan University, Wuhan, Hubei China; 4 Central Laboratory, Renmin Hospital of Wuhan University, Wuhan, Hubei China; 5 Department of Anesthesiology, Second Affiliated Hospital of Zhejiang University School of Medicine, Hangzhou, China

**Keywords:** Stroke, Neuroinflammation, CD4 T cells, Akt, MTOR, PTEN

## Abstract

**Background:**

Inhibition of CD4 T cells reduces stroke-induced infarction by inhibiting neuroinflammation in the ischemic brain in experimental stroke. Nevertheless, little is known about its effects on neuronal survival signaling pathways. In this study, we investigated the effects of CD4 T cell deficits on oxidative stress and on the Akt/mTOR cell signaling pathways after ischemic stroke in mice.

**Methods:**

MHC II gene knockout C57/BL6 mice, with significantly decreased CD4 T cells, were used. Stroke was induced by 60-min middle cerebral artery (MCA) occlusion. Ischemic brain tissues were harvested for Western blotting.

**Results:**

The impairment of CD4 T cell production resulted in smaller infarction. The Western blot results showed that iNOS protein levels robustly increased at 5 h and 24 h and then returned toward baseline at 48 h in wild-type mice after stroke, and gene KO inhibited iNOS at 5 h and 24 h. In contrast, the anti-inflammatory marker, arginase I, was found increased after stroke in WT mice, which was further enhanced in the KO mice. In addition, stroke resulted in increased phosphorylated PTEN, Akt, PRAS40, P70S6, and S6 protein levels in WT mice, which were further enhanced in the animals whose CD4 T cells were impaired.

**Conclusion:**

The impairment of CD4 T cell products prevents ischemic brain injury, inhibits inflammatory signals, and enhances the Akt/mTOR cell survival signaling pathways.

## Background

Neuroinflammation plays critical roles in secondary brain injury after stroke. Immediately after stroke, neuronal injury in the ischemic core results in releases of pro-inflammatory factors such as ATP, glutamate, and pro-inflammatory cytokines, into the ischemic tissues, which stimulate the activation of resident microglia, which transform into macrophages [1–4]. The activated microglia/macrophages and the released inflammatory factors result in the opening of the blood-brain barrier (BBB) [5] and the recruitment of monocytes, neutrophils, T cells, and T cell subsets, including CD4 and CD8 T cells, into the ischemic tissue, further enlarging ischemic brain injury [6–12]. We previously reported that the impairment of CD 4 T cell production in MHC II gene KO mice reduces brain injury in mice after focal cerebral ischemia [7]. Nevertheless, the underlying protective mechanisms of CD4 deficits against stroke are poorly understood.

Neuroinflammation is closely related with oxidative stress, which is reflected by iNOS and arginase I protein expression levels [13]. Overactivation of iNOS produces free radicals such as NO and NO-derived products, which promote neuroinflammation. Arginase I is a well-recognized marker of the anti-inflammatory M2 macrophage phenotype [14, 15]. How CD4 T cells affect iNOS and arginase I in the ischemic brain remains elusive. In addition, we and others have reported that stroke results in the activation of the Akt/mTOR neuronal survival signaling pathways [16–18]. In the PI3K/Akt pathway, PTEN is a phosphatase that dephosphorylates Akt. Phosphorylated, active Akt blocks apoptosis by phosphorylating a number of downstream substrates, including the forkhead transcription factor FKHR (FOXO1), GSK3b, PRAS40, and mTOR. We showed that PRAS40 plays a pivotal role in linking the Akt and the mTOR pathways. Once active, mTOR causes further phosphorylation of downstream proteins, such as the p70S6 ribosomal protein kinase1 (S6K1), which regulates protein translation and cell growth [19]. We have reported that the overexpression of Akt and PRAS40 prevents brain injury, while PRAS40 gene KO and mTOR inhibition results in larger brain infarction [17, 18]. Nevertheless, how CD4 T cell deficit affects the Akt/mTOR cell signaling pathway has not been studied.

In this study, we investigated the effect of CD4 T cell deficit on oxidative stress responses and the Akt/mTOR pathways in a mouse stroke model with transient MCA suture occlusion by using MHC II gene KO mice with dramatically reduced CD4 T cells.

## Methods

### Animals

The study protocols were approved by the Stanford Institutional Animal Care and Use Committee. Animal experiments were conducted according to the NIH Guidelines for Care and Use of Laboratory Animals. Mice were housed under a 12:12-h light-dark cycle and allowed free access to food and water before the experiment.

### Focal cerebral ischemia

Twenty-six C57B6 wild-type (WT) and 24 MHC II gene knockout (KO) (strain name: B6.129S2-*H2*^*dlAb1*^-Ea/J) mice were used. All animals were purchased from the Jackson Laboratory, who had confirmed a dramatic reduction of CD4 T cells in the MHC II gene KO mice. Animals were anesthetized with 3% isoflurane and maintained by 1.5–2% isoflurane oxygen-enriched air (fraction of inspired oxygen [FiO_2_]: 40%) by a face mask in both male MHC II gene KO mice and WT mice (25 to 30 g), as we previously reported [7, 18]. Rectal temperature was maintained at 37 ± 0.5 °C with a heating pad (Harvard Apparatus, Hollister, MA). Transient focal ischemia was induced by 60-min middle cerebral artery occlusion (MCAO), as previously described. In brief, we introduced a silicone-coated 6-0 monofilament into the left external carotid artery (ECA) and advanced it from the carotid bifurcation to occlude the MCA. Isoflurane was discontinued after suture insertion, and the mice were revived. Mice were re-anesthetized 60 min later, and the filament was withdrawn. Sham-operated mice underwent the same procedure, except that the monofilament was not inserted.

### Measurement of cerebral infarction

Three days after stroke, the brains were removed and cleaved into four coronal sections with a 2.0-mm slice interval using a rodent brain slicer matrix (Zivic Instruments, Pittsburgh, PA), as we reported [7, 18]. Sections were incubated in 2% 2,3,5-triphenyletrazolium chloride (TTC; #T8877, Sigma-Aldrich, St. Louis, MO). Infarct volume (percent of hemispheric volume) was determined by one blinded observer and corrected for edema using the NIH Image J program (Image J 1.37v; Wayne Rasband, available through NIH) as described previously.

### Western blotting

To study the effects of CD4 impairment on protein levels, mice were euthanized at 5 h, 24 h, and 48 h after reperfusion by an overdose of isoflurane. The ischemic hemispheres were collected, and whole cell proteins were extracted. Western blot was performed, as described, in our previous study [20–22]. Table 1 lists the primary antibodies used.

**Table 1:** Antibodies and their concentrations, manufacturers, and applications for Western blot

Antibodies	Source	Dilutions	Manufacturer	Catalog no.
iNOS	Mouse	1:10,000	BD Biosciences	610431
Arginase I	Goat	1:100	Santa Cruz	18354
p-PTEN (Ser380)	Rabbit	1:1000	Cell Signaling	9551
p-Akt (Ser473)	Rabbit	1:100/1:1000	Cell Signaling	9271
p-PRAS40 (Thr246)	Rabbit	1:100/1:1000	Cell Signaling	2997
p-P70S6K	Rabbit	1:1000	Cell Signaling	9205
p-S6	Rabbit	1:1000	Cell Signaling	4857
β-Actin	Rabbit	1:1000	Cell Signaling	4967

In each lane, 30 μg proteins were subjected to sodium dodecyl sulfate–polyacrylamide gel electrophoresis using 4–15% Ready Gel (Bio-Rad Laboratories, Hercules, CA, USA) for 1.5 h. Protein bands were then transferred to polyvinylidene fluoride membranes (Millipore, Bedford, MA, USA) for 1 h, then blocked with 5% nonfat dry milk (Bio-Rad Laboratories) in PBS/0.05% Tween-20. The membranes were then incubated in the primary antibodies overnight at 4 °C, followed by horseradish peroxidase (HRP)-conjugated secondary antibody (anti-rabbit 1: 2000, Cell Signaling Technology) or anti-mouse IgG for 1 h. Subsequently, immunoreactive bands were visualized with enhanced chemiluminescence (ECL Kit, Santa Cruz Biotechnology, USA) and exposed to radiographic film to detect the goal protein bands. The membranes were incubated with anti-β-actin antibodies as an even protein loading control. Membranes were scanned using Typhoon trio (GE Healthcare, Waukesha, WI, USA). Optical band densities were analyzed and normalized with β-actin using Image J software.

### Statistical analysis

The data were presented as mean ± standard deviation (SD). Means were compared by two-tailed unpaired *t* test and one-way analysis of variance (ANOVA) for comparison of multiple samples with Prism5 software (GraphPad, Software for Science, San Diego, CA, USA). Differences were considered statistically significant for *P* value < 0.05.

## Results

### CD4 T cell deficit is neuroprotective in stroke

**Fig. 1:**
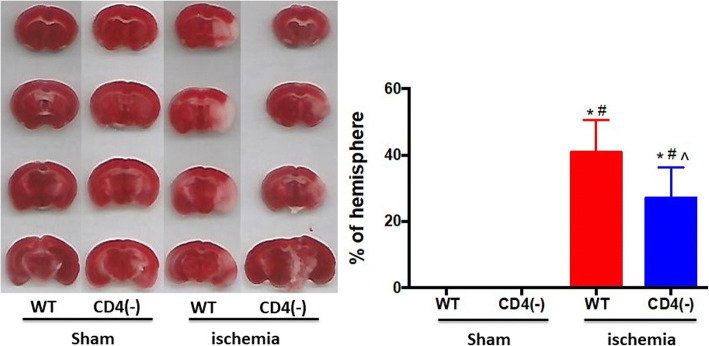
The impairment of CD4 T cell production resulted in smaller infarction. Representative TTC staining of infarction is shown. The bar graph represents the statistical results of infarct sizes. *N* = 6/group. *P < 0.05, vs WT-sham; ^#^*P* < 0.05, vs CD4(−)-sham; ^*P* < 0.05, vs WT. WT, wild type; CD4(−), CD4 deficit

As consistent with our previous report [7], CD4 T cell deficits resulted in smaller infarction (Fig. 1).

### CD4 T cell deficits inhibited iNOS but enhanced arginase I levels after stroke

**Fig. 2:**
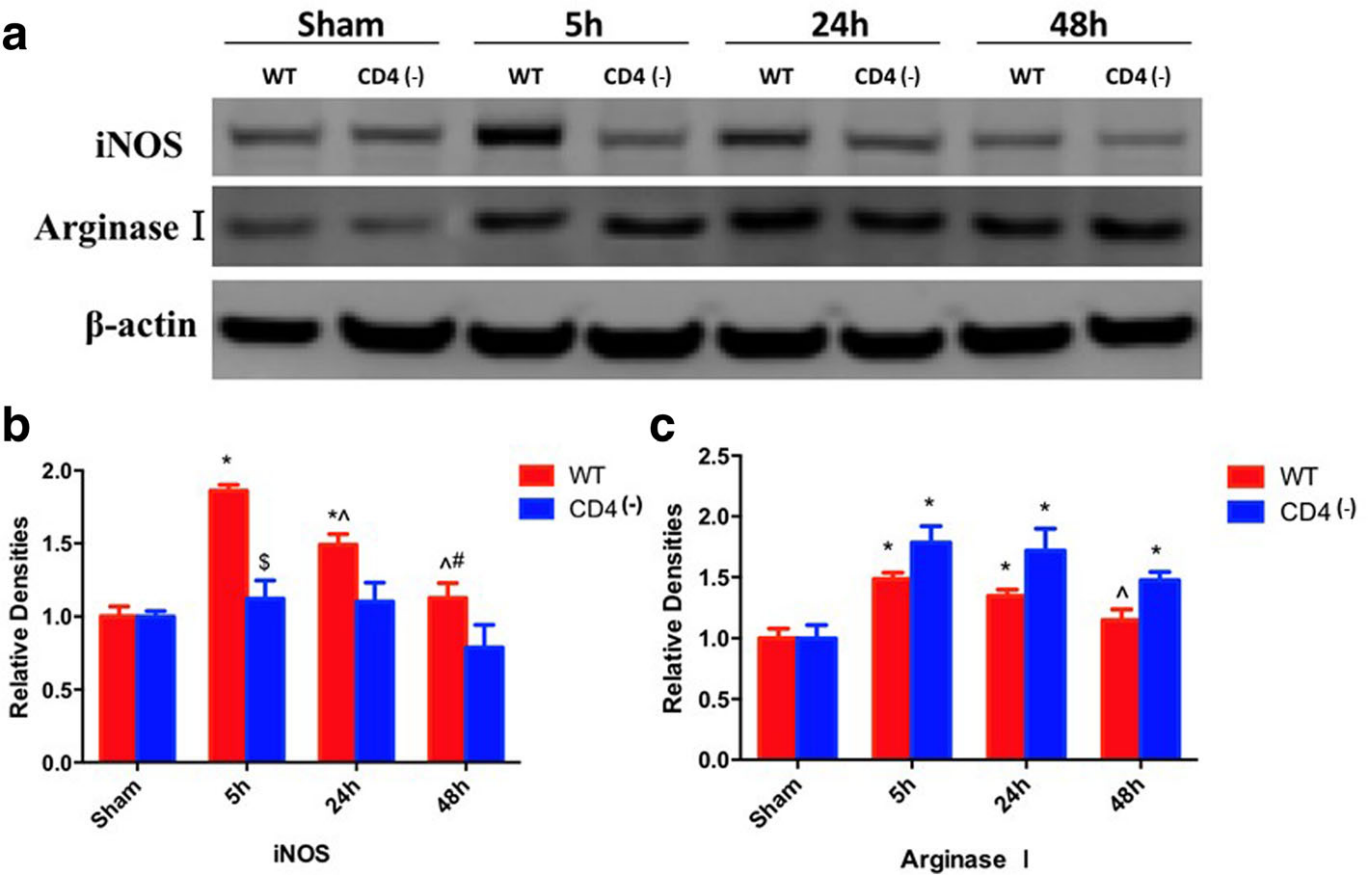
**a**–**c** The effects of CD4 T cell impairment on iNOS and arginase 1 protein levels. Representative protein bands from Western blot are shown. The bar graph shows the statistical results of protein levels; the values are fold changes compared with the sham. β-Actin was probed to show even protein loading. One-way ANOVA was used to compare the statistical difference of all mice between the WT group and the KO group. *N* = 3–4/group, *P < 0.05, vs sham; ^*P* < 0.05, vs 5 h; ^#^*P* < 0.05, vs 24 h; ^$^*P* < 0.05, vs WT. WT, wild type; CD4(−), CD4 deficit

We determined the iNOS and arginase I protein levels in WT and MHC II KO mice by using Western blot (Fig. 2). Brain tissues were collected at 5 h, 24 h, and 48 h after stroke. The results showed that the iNOS protein levels were dramatically increased at 5 h after stroke, and then gradually decreased from 24 h and 48 h (Fig. 2a). Nevertheless, the iNOS protein levels showed an increase after stroke in KO mice compared with the sham animals, and its level is significantly lower than in WT mice at 5 h (Fig. 2a).

Arginase I protein markers were significantly increased at 5 h and 24 h, but not at 48 h, compared with sham in WT mice. However, CD4 deficits further resulted in higher arginase I protein levels at 5 h and 24 h, as well as 48 h after stroke, compared with WT mice (Fig. 2b).

### PRAS40 protein levels were enhanced in the KO mice but not in WT mice

**Fig. 3:**
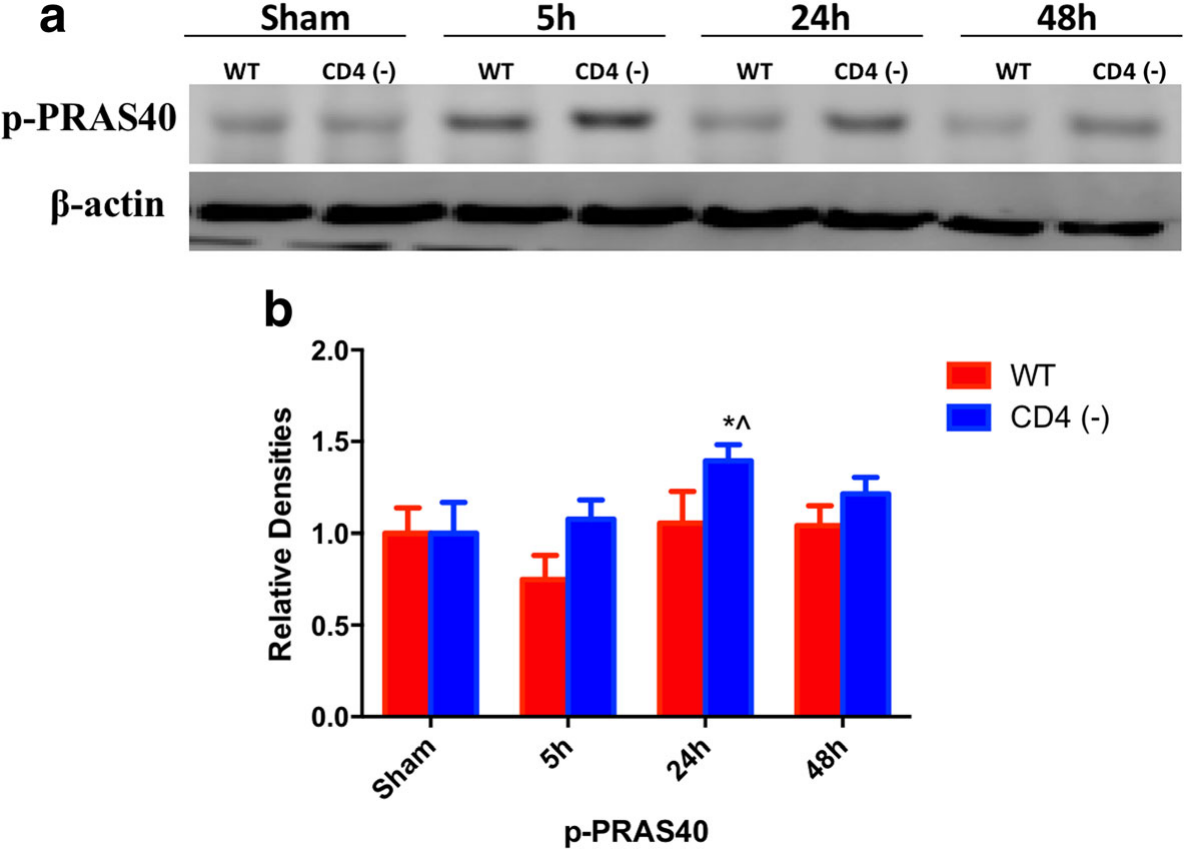
**a**, **b** The effects of CD4 T cell impairment on p-PRAS40 protein levels. Representative protein bands from Western blot are shown. The bar graph shows the statistical results of protein levels. β-Actin was probed to show even protein loading. *N* = 3/group, *P < 0.05, vs sham; ^*P* < 0.05, vs 5 h. WT, wild type; CD4(−), CD4 deficit

PRAS40 is a pivotal molecule, linking the Akt and mTOR pathways. Western blot results showed that P-PRAS40 protein levels had no significant changes after stroke, from 5 h to 48 h in WT mice, but CD4 T cell deficits significantly enhanced its levels at 24 h after stroke (Fig. 3).

### The effect of CD4 T cell deficits on the Akt and mTOR pathway

**Fig. 4:**
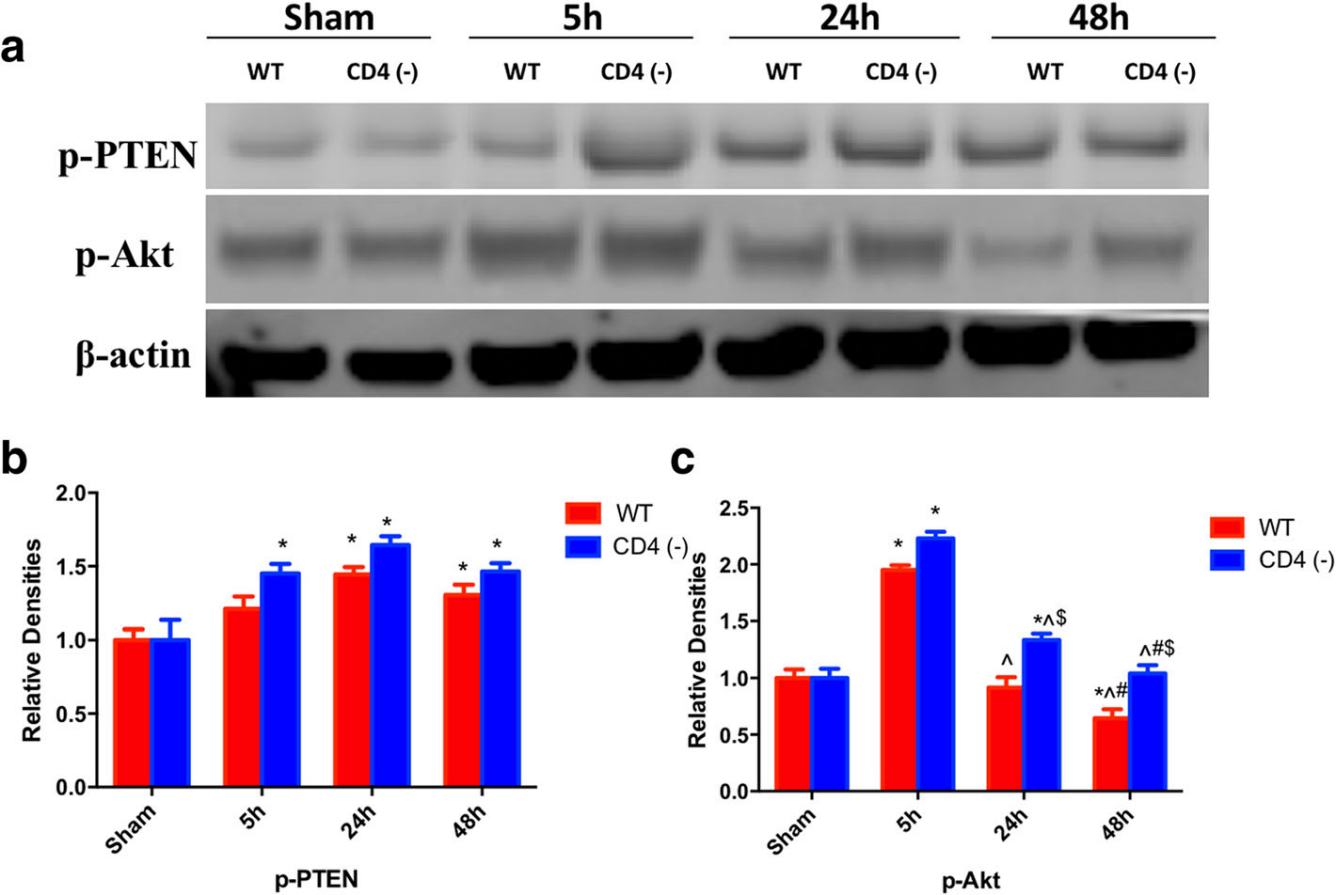
**a**–**c** Comparison of p-PTEN and p-Akt protein levels in WT and KO mice. Representative protein bands from Western blot are shown. The bar graph shows the statistical results of protein levels. β-Actin was probed to show even protein loading *N* = 3–4/group, *P < 0.05, vs sham; ^*P* < 0.05, vs 5 h; ^#^*P* < 0.05, vs 24 h; ^$^*P* < 0.05, vs WT. WT, wild type; CD4(−), CD4 deficit

We then measured the protein levels in the Akt and mTOR pathways. The results show that p-PTEN and p-Akt protein levels were significantly increased after stroke in WT mice, and the impairment of CD4 T cells resulted in higher protein levels in the MHC II KO mice, though no significant differences were detected between the WT and KO mice (Fig. 4).

**Fig. 5:**
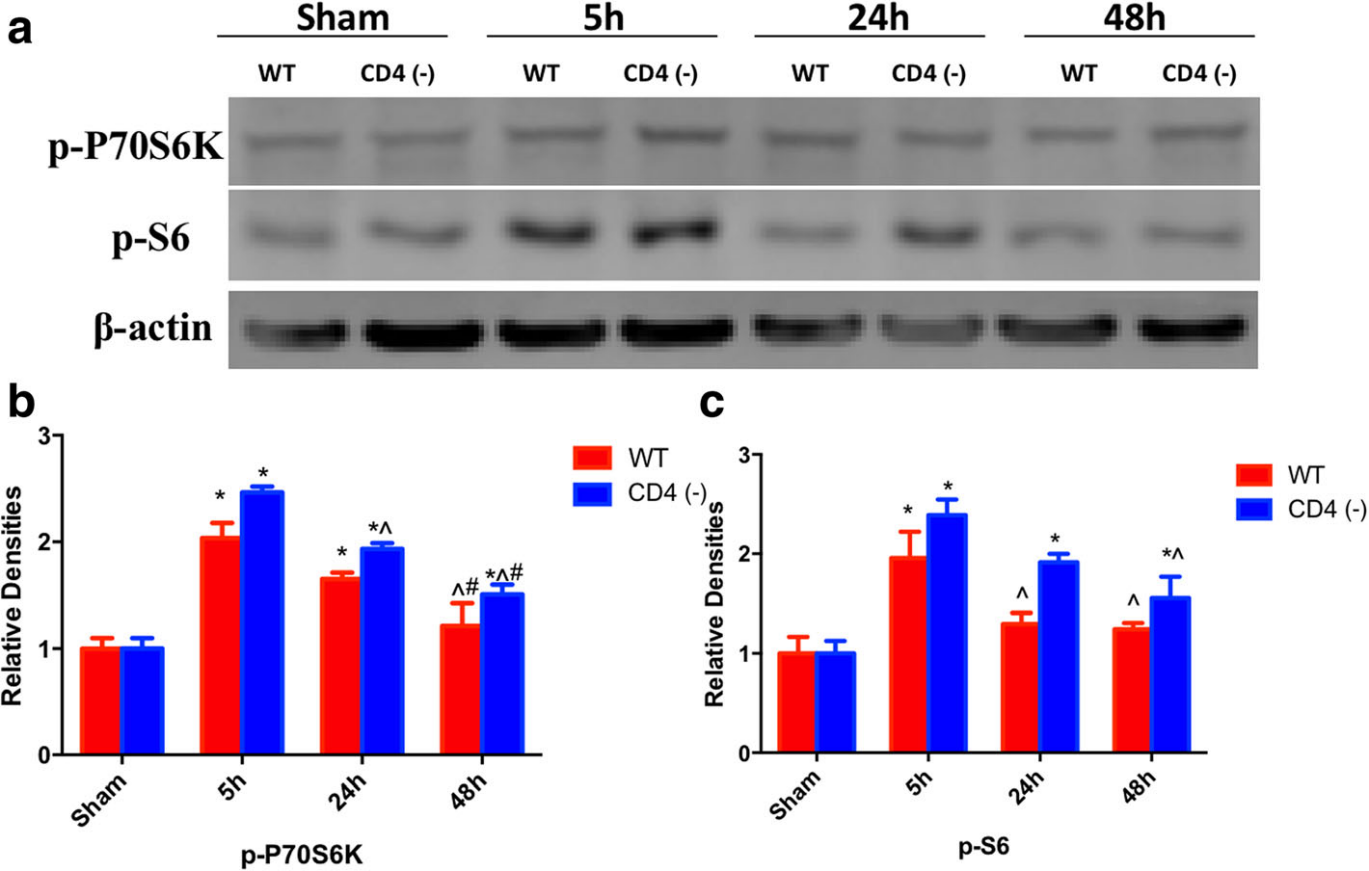
**a**–**c** Western blot results of p-P70S6K and p-S6 protein levels in the ischemic brain in WT and KO mice. Representative protein bands from Western blot are shown. The bar graph shows the statistical results of protein levels. β-Actin was probed to show even protein loading. *N* = 3–4/group, *P < 0.05, vs sham; ^*P* < 0.05, vs 5 h; ^#^*P* < 0.05, vs 24 h. WT, wild type; CD4(−), CD4 deficit

Similarly, P-P70S6K protein levels were significantly increased at 5 h and 24 h, but not at 48 h in WT mice. CD4 T cell deficits further enhanced its protein levels, although a significant difference between the WT and KO mice was not reached (Fig. 5a).

The P-S6 protein levels were significantly increased at 5 h, but not at 24 h and 48 h after stroke. Nevertheless, their levels were significantly increased at all the measured time points, from 5 h to 48 h, in the KO mice (Fig. 5b).

## Discussion

In this study, we have shown some unprecedented results, suggesting that the protective effects of CD4 T cell deficits against stroke is linked with inhibited oxidative stress and enhanced cell signaling survival pathways. First, iNOS protein levels are significantly inhibited while the anti-inflammatory marker arginase I protein levels are enhanced in animals with CD4 T cell deficit. Second, CD4 T cell deficit does not significantly alter p-PTEN protein levels after stroke compared with WT mice, but it significantly enhanced P-Akt protein levels. Third, although stroke did not significantly alter the phosphorylation of PRAS40 between the Akt and the mTOR pathways, CD4 T cell deficit results in significantly higher levels of P-PRAS40 at 24 h compared with WT mice. Fourth, stroke results in increased P-P70S6K and P-S6K protein levels after stroke in WT mice, but CD4 T cell deficit significantly promoted their expression in the KO mice. Taken together, we provide solid evidence that the protective effect of CD4 T cell deficits in stroke is linked with the inhibited pro-inflammatory and oxidative responses, and with the enhanced activities of the Akt/mTOR pathways.

The detrimental effects of CD4 T cells in stroke-induced brain injury have been repeatedly confirmed. We and others have reported that CD4 T cells infiltrate into the ischemic brain, suggesting that CD4 T cells are involved in neuroinflammation induced by stroke [6, 8, 12, 23]. In addition, CD4 T cell deficit reduces infarction [12]. As consistent with other studies, we previously reported that CD4 T cell deficit in MHC II KO mice results in smaller infarction in vivo, and in vitro co-culture of lymphocytes without CD4 T cells kills less neurons than lymphocytes with CD4 T cells [7]. Despite these solid studies, how CD4 T cells affect brain injury remains poorly understood. We speculate that CD4 T cells exacerbate brain injury by activating macrophages, as macrophages outnumber any other inflammatory cells in the ischemic brain; thus, macrophages may be the final effectors for neuronal injury induced by inflammatory response after stroke. As macrophages are polarized into pro-inflammatory M1 and anti-inflammatory M2 macrophages, which can be marked by iNOS and arginase I [24–27], respectively, we examined their protein levels after stroke. As consistent with our expectation, CD4 T cell deficit inhibited iNOS protein levels while it enhanced arginase I protein levels, suggesting that CD4 T cell deficit resulted in an inhibited pro-inflammatory response while it enhanced the anti-inflammatory action.

We and others have extensively studied the neuroprotective effects of the Akt/mTOR pathways in stroke. Phosphorylated Akt, PTEN, mTOR, P70S6K, and S60 protein levels are increased after stroke in the ischemic brain, suggesting a stimulating effect of stroke on these protective proteins. We have also reported that the overexpression of Akt- or mTOR-related downstream molecules inhibits brain injury, while inhibition of Akt and mTOR exacerbates ischemic damage [16–18, 28]. PRAS40 is a link between the Akt and the mTOR pathway, and our previous study suggests that PRAS40 KO enlarges infarction while the overexpression of PRAS40 by lentiviral vector gene transfer inhibits brain injury. KO has detrimental effects after stroke [18]. In our current study, we show evidence that the protective effects of CD4 T cell deficit are strongly linked with the Akt/mTOR pathways, as both P-Akt levels, and P-P70S6K and P-S6, two downstream proteins in the mTOR pathway, are enhanced in the KO mice. These effects should be distinguished from other reports showing that the Akt/mTOR pathways play important roles in T cell function [29, 30], as our purpose was to examine how CD4 T cell deficits affect the Akt/mTOR pathways in bran tissues, rather than how the Akt/mTOR pathways affect CD4 T cells.

## Conclusion

The impairment of CD 4 cell production protects against acute brain injury, inhibited neuroinflammation and oxidative stress, and promoted the Akt/mTOR survival cell signaling pathways.

## Abbreviations

CD4: Cluster of differentiation 4
iNOS: Inducible nitric oxide synthase
KO: Knockout
P-P70S6K: 70-kDa ribosomal S6 kinase
PRAS40: Proline-rich Akt substrate of 40 kDa
P-S6: Ribosomal protein *S6* kinase
PTEN: Phosphatase and tensin homolog
WT: Wild type
